# Atherogenic lipid profile is a feature characteristic of patients with early rheumatoid arthritis: effect of early treatment – a prospective, controlled study

**DOI:** 10.1186/ar1952

**Published:** 2006-04-28

**Authors:** Athanasios N Georgiadis, Eleni C Papavasiliou, Evangelia S Lourida, Yannis Alamanos, Christina Kostara, Alexandros D Tselepis, Alexandros A Drosos

**Affiliations:** 1 Department of Internal Medicine, Medical School, University of Ioannina, 45110 Greece; 2 Department of Chemistry, Laboratory of Biochemistry, University of Ioannina, 45110 Greece; 3 Department of Hygiene and Epidemiology, Medical School, University of Ioannina, 45110 Greece; 4 Laboratory of Biochemistry, University Hospital of Ioannina, 45500 Greece

## Abstract

We investigated lipid profiles and lipoprotein modification after immuno-intervention in patients with early rheumatoid arthritis (ERA). Fifty-eight patients with ERA who met the American College of Rheumatology (ACR) criteria were included in the study. These patients had disease durations of less than one year and had not had prior treatment for it. Smokers or patients suffering from diabetes mellitus, hypothyroidism, liver or kidney disease, Cushing's syndrome, obesity, familiar dyslipidemia and those receiving medications affecting lipid metabolism were excluded from the study. Sixty-three healthy volunteers (controls) were also included. Patients were treated with methotrexate and prednisone. Lipid profiles, disease activity for the 28 joint indices score (DAS-28) as well as ACR 50% response criteria were determined for all patients. The mean DAS-28 at disease onset was 5.8 ± 0.9. After a year of therapy, 53 (91.3%) patients achieved the ACR 20% response criteria, while 45 (77.6%) attained the ACR 50% criteria. In addition, a significant decrease in the DAS-28, C-reactive protein (CRP) and erythrocyte sedimentation rate (ESR) were observed. ERA patients exhibited higher serum levels of total cholesterol (TC), low-density lipoprotein cholesterol (LDL-C) and triglycerides, whereas their serum high-density lipoprotein cholesterol (HDL-C) levels were significantly lower compared to controls. As a consequence, the atherogenic ratio of TC/HDL-C as well as that of LDL-C/HDL-C was significantly higher in ERA patients compared to controls. After treatment, a significant reduction of the atherogenic ratio of TC/HDL-C as well as that of LDL-C/HDL-C was observed, a phenomenon primarily due to the increase of serum HDL-C levels. These changes were inversely correlated with laboratory changes, especially CRP and ESR. In conclusion, ERA patients are characterized by an atherogenic lipid profile, which improves after therapy. Thus, early immuno-intervention to control disease activity may reduce the risk of the atherosclerotic process and cardiovascular events in ERA patients.

## Introduction

Rheumatoid arthritis (RA) is a chronic systemic disease affecting primarily the synovium, leading to joint damage and bone destruction [1]. RA causes significant morbidity as a result of synovial inflammation, joint destruction and associated disability [2]. Epidemiological studies have shown an increased premature mortality in patients with RA compared with the general population [3-7]. Several investigators reported an excess of cardiovascular morbidity and mortality among RA patients. In active RA, the majority of cardiovascular deaths result from accelerated atherosclerosis [2,8,9]. Risk factors for atherosclerotic events and cardiovascular disease include male sex, increased age, elevated plasma total cholesterol (TC) and low-density lipoprotein cholesterol (LDL-C), decreased high-density lipoprotein cholesterol (HDL-C), high blood pressure, smoking and diabetes mellitus [10-13]. Approximately 50% of atherosclerotic coronary artery disease (CAD) in the community occurs in the absence of traditional risk factors [14].

In general, and with some variations between different studies, the lipid profile of patients with active or untreated RA is primarily characterized by a decrease in serum levels of HDL-C whereas contrasting results have been published on the serum levels of TC and LDL-C [15-20]. Importantly, the reduction in HDL-C has as a consequence the increase in the TC/HDL-C ratio [15]. This ratio represents an atherogenic index, which is an important prognostic marker for cardiovascular disease [15]. Indeed, the risk of myocardial infarction increases considerably when this ratio is higher than five, and it should ideally be four or less [15,16]. The serum TC and HDL-C levels in RA are inversely correlated with disease activity [15], suggesting a potential role for inflammation in the atherogenic profile and the higher atherosclerotic risk observed in RA [17].

The cholesterol ester transfer protein (CETP) has a central role in HDL metabolism and in the regulation of HDL-C levels in serum. CETP exchanges cholesterol esters with triglycerides between HDL and apolipoprotein B-containing lipoproteins and thus significantly contributes to the reverse cholesterol transport pathway. High levels of CETP activity lead to a reduction in HDL-C levels and an atherogenic lipoprotein profile [21,22]. Thus, mutations in the CETP gene associated with CETP deficiency are characterized by high serum HDL-C levels and reduced cardiovascular risk [23].

Our knowledge about the effect of treatment on the lipid profile of patients with RA is limited and only cross-sectional and short term uncontrolled studies have been performed [15,17-20]. In addition, studies on lipid profile and CETP activity, as well as studies on the effect of therapy on these parameters in early RA (ERA) patients, are scarce. On the other hand, atherosclerosis is a chronic process and only long-term changes of the lipid profile might affect cardiovascular disease. Therefore, we undertook a prospective, controlled study to investigate the lipid profile and CETP activity in ERA patients as well as the long-term effects of immuno-intervention on these parameters in patients with active disease.

## Materials and methods

### Patients

#### Inclusion criteria

Fifty-eight consecutive, unselected patients who were referred to the outpatient rheumatology clinic between January 2004 and September 2005 were investigated. All patients fulfilled the American College of Rheumatology (ACR) 1987 criteria for RA [24], had early disease with disease duration of less than one year without prior use of disease modifying antirheumatic drugs (DMARDs) and or systemic steroids.

#### Exclusion criteria

Smokers or patients suffering from conditions that affect the lipid profile, such as diabetes mellitus, hypothyroidism, liver or kidney disease, Cushing's syndrome, obesity (body mass index >30) and a history of familial dyslipidemia, were excluded. In addition, patients receiving medications affecting lipid metabolism, such as lipid-lowering drugs, beta-blockers, oral contraceptives, estrogen, progestin, thyroxin and vitamin E, were excluded from the study.

Sixty-three apparently healthy, non-smoking volunteers also participated in the study and were used as a control group. These subjects were selected from blood donors during the last two months of the patients' selection period and fulfilled the same exclusion criteria reported for the patient group. None of the subjects participating in the control group had a history of CAD. The control group was proportionally matched for age and sex to the patient group. All controls reported no significant changes in their body weight for at least three months before entry to the study. All patients and controls gave informed consent and the study protocol was approved by the Institutional Ethics Committee.

### Study design

Patients were treated with methotrexate (MTX; 0.2 mg/kg/week; mean ± standard deviation 15.5 ± 1.3) and prednisone (7.5 mg/day). The dose of MTX remained stable during the study while the dose of prednisone was tapered according to a patient's clinical response. Disease activity was assessed by measuring the disease activity for 28 joint indices score (DAS-28) [25], while the clinical response was evaluated according to the ACR 50% response criteria [26]. All patients were followed up every month for the first three months, and every three months thereafter. During the follow up period, a questionnaire concerning changes in dietary habits was carefully fulfilled by all patients. The body weight was also measured appropriately in each visit.

### Blood sampling and laboratory monitoring

Overnight fasting blood samples were obtained at baseline and after 12 months follow-up from both ERA patients and the control group. Serum lipids were determined within six hours of blood sampling. TC, triglycerides and HDL-C were determined on an Olympus AU560 Clinical Chemistry analyser (Hamburg, Germany) as previously described [27]. LDL-C was estimated using the Friedewald formula [28]. Non-HDL-C levels were estimated by subtracting HDL-C from TC. Serum apolipoproteins B and A-I (apoB and apoA-I, respectively) were measured by immunonephelometry with the aid of a Behring Nephelometer BN100 and reagents (antibodies and calibrators) from Behring Diagnostics GmbH (Liederbach, Germany). C-reactive protein (CRP) and IgM rheumatoid factor were measured by nephelometry. Erythrocyte sedimentation rate (ESR) was measured by the modified Westergren method. In addition, complete blood count with differential, as well as serum glucose, liver and kidney function tests and urinalysis, were performed at each patient visit until the end of the study. CETP activity was measured by a fluorometric assay, using a commercially available kit (Roar Biomedical, Inc., New York, NY, USA). Briefly, 2 μl of plasma diluted 1:1 with sample buffer (10 mmol/l Tris, 150 mmol/l NaCl, and 2 mmol/l EDTA, pH 7.4) were used as the source of CETP. The assay was performed for 1 hour at 37°C and the CETP activity was determined by the increase in fluorescence intensity measured in a fluorescence spectrometer at an excitation wavelength of 465 nm and emission wavelength of 535 nm [29].

### Statistical analysis

All data were analyzed with the STATISTICA 5.1 program. Comparisons between groups were conducted using the *t *test. Correlation between variables was examined using the Pearson's correlation coefficient. A *p *value < 0.05 was considered statistically significant.

## Results

During the selection period (January 2004 to September 2005), a total of 72 patients were recruited. Of these, 14 were excluded; 7 who were current smokers, 2 with diabetes mellitus, 4 who had hypertension treated with propranolol and diuretics and 1 who had hypothyroidism. Thus, 58 patients were included in the study. None of the patients reported a modification of dietary habits or experienced significant changes in body weight during the study. The clinical characteristics and lipid profiles of patients before and after therapy and normolipidemic controls are described in Table 1. There were 44 women and 14 men with a mean age of 53.6 ± 15.3 years and mean disease duration of 0.8 ± 0.3 years. Fifty-three (91.3%) of the patients achieved the ACR 20% response criteria, while 45 (77.6%) attained the ACR 50% criteria. Forty-three (74.13%) patients had a good response, while 11 (18.96%) had a moderate response, according to the European League Against Rheumatism criteria [30]. The mean dose of prednisone after one year of treatment was 4.6 ± 1.8 mg/day. No significant side effects were noted during the study, the drugs being well tolerated, while two patients were lost to follow-up.

ERA patients exhibited mild but significant higher baseline serum levels of TC, LDL-C, nonHDL-C, triglycerides and apoB (Table 1). Importantly, the serum HDL-C and apoA-I levels were significantly lower compared to controls. As a consequence, the atherogenic ratio of TC/HDL-C as well as that of LDL-C/HDL-C was significantly higher in ERA patients compared to controls.

After 12 months of therapy, a significant decrease in the DAS-28 and ESR values as well as in the CRP levels were observed (Table 1). Furthermore, post treatment levels of HDL-C were significantly higher compared to the baseline values. By contrast, the serum levels of LDL-C and nonHDL-C were not significantly altered after treatment. Due to the significant increase in HDL-C levels, the post treatment levels of TC were significantly elevated (Table 1). Importantly, the atherogenic ratios TC/HDL-C and LDL-C/HDL-C were significantly reduced after treatment, a phenomenon primarily due to an early treatment-induced increase in the serum levels of HDL-C (Table 1). Finally, no changes in the post treatment serum levels of triglycerides were noted.

**Figure 1:**
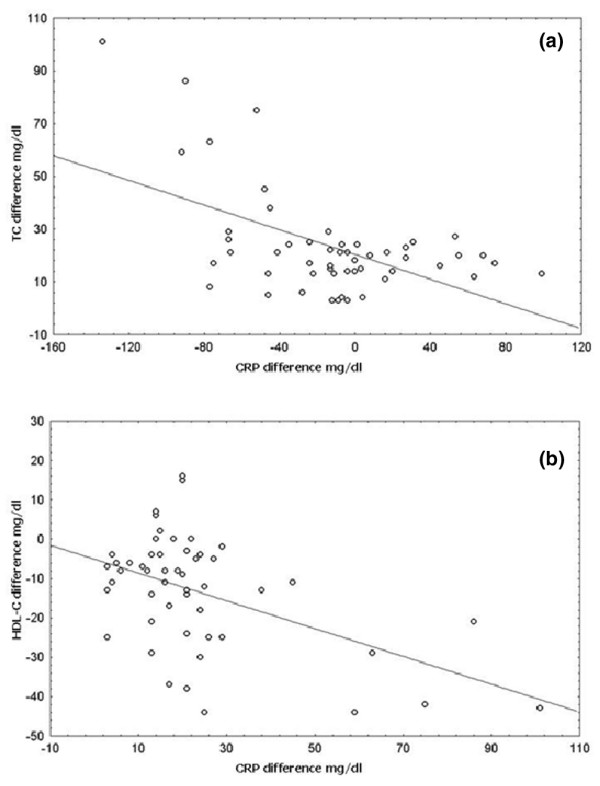
Correlation of total cholesterol (TC) and high-density lipoprotein cholesterol (HDL-C) with C-reactive protein (CRP). Inverse correlation of CRP differences with **(a) **TC and **(b) **HDL-C differences (r = -0.54, *p *< 0.0001 and r = -0.59, *p *< 0.0001, respectively).

**Figure 2:**
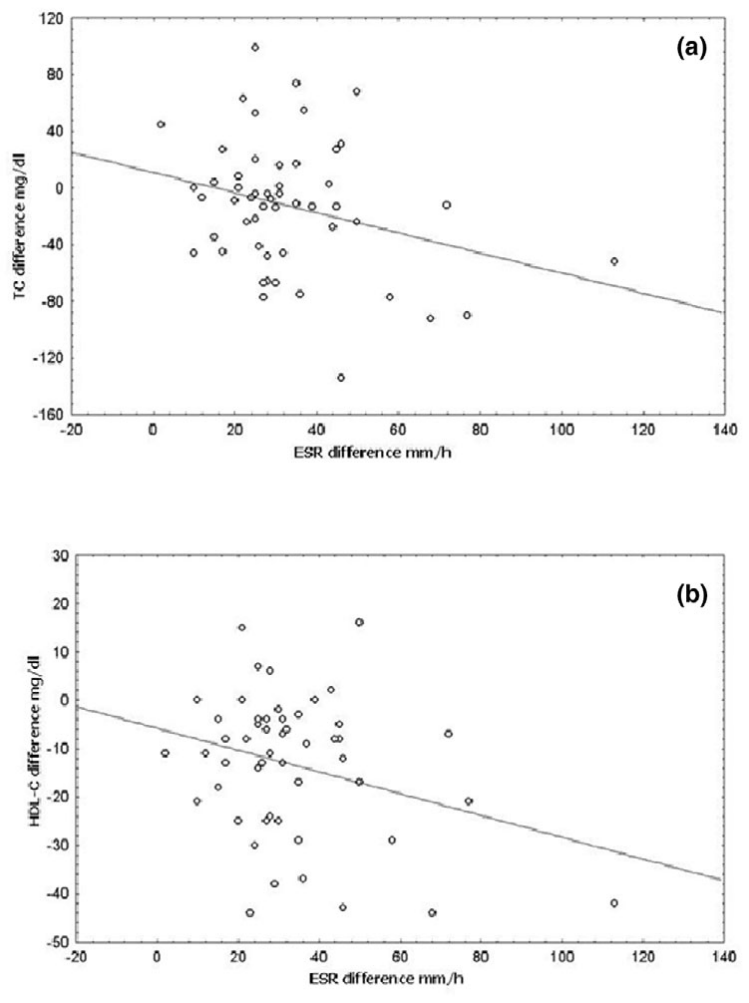
Correlation of total cholesterol (TC) and high-density lipoprotein cholesterol (HDL-C) with erythrocyte sedimentation rate (ESR). Inverse correlation of ESR differences with **(a) **TC and **(b) **HDL-C differences (r = -0.28, *p *< 0.04 and r = -0.30, *p *< 0.03, respectively).

It should be noted that the increase in TC and HDL-C serum levels induced by early immuno-intervention was inversely correlated with the reduction in the serum CRP levels and ESR values (Figure 1a,b and Figure 2a,b). Finally, no correlation between the DAS-28 values and the above lipid parameters were observed (data not shown).

**Figure 3:**
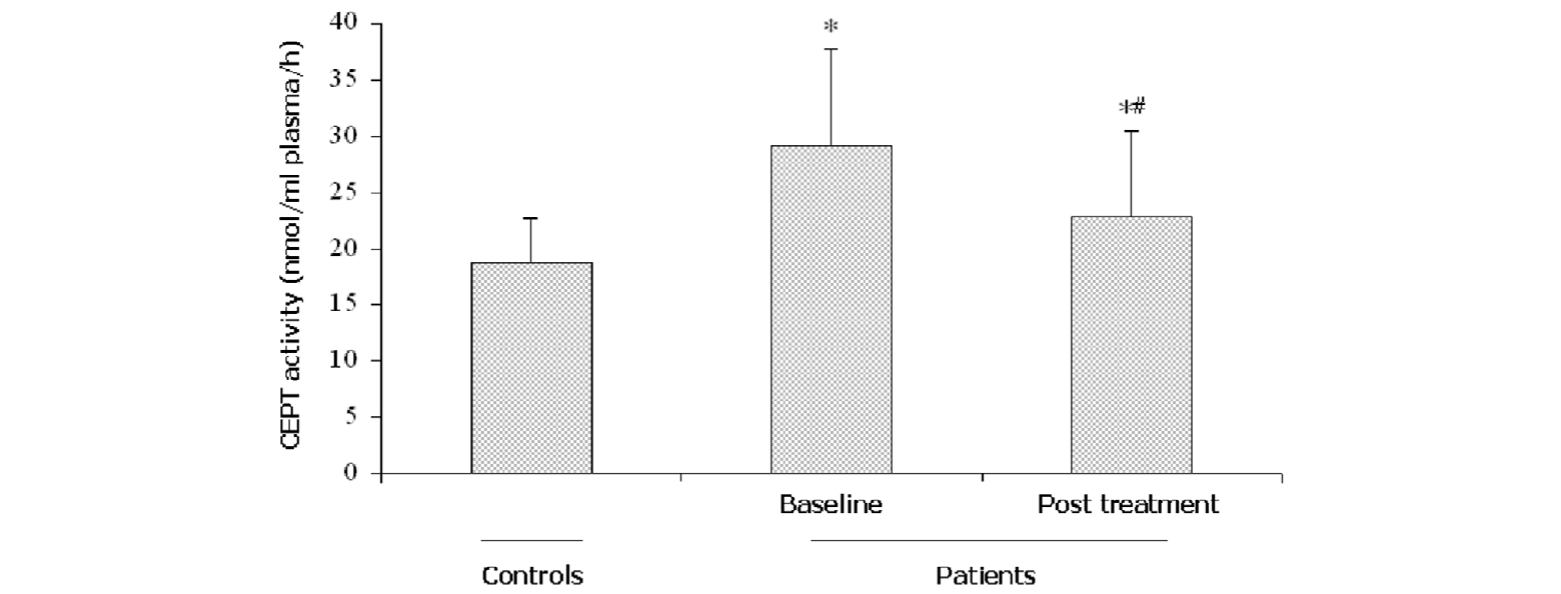
Cholesterol ester transfer protein (CETP) activity of controls and early rheumatoid arthritis patients before and after therapy. Data are expressed as means ± standard deviation. *p < 0.001 compared with controls; ^#^*p *< 0.05 compared with pretreatment values.

The most important observation of the present study is the increase of serum HDL-C levels induced by immuno-intervention in ERA patients. In an effort to investigate the mechanism for the HDL-C increase, we determined the CETP activity in controls as well as in ERA patients before and after one year of therapy. The baseline values of CETP activity in ERA patients were significantly higher compared to controls, whereas they were significantly reduced after one year of therapy (Figure 3). It should be noted that no significant alterations were observed in the CETP activity of controls after one year of follow-up (19.0 ± 4.1).

## Discussion

Our objective was to determine the lipid profile of ERA patients and to investigate whether this could be influenced by treating RA in the early stages of disease. According to our results, patients with early active RA before therapy exhibited an atherogenic lipid profile characterized by an increase of TC, LDL-C, nonHDL-C and triglyceride serum levels and a reduction in serum HDL-C levels. Thus, an increase in the atherogenic ratio of TC/HDL-C or LDL-C/HDL-C is observed in ERA patients, suggesting that these patients are possibly exposed to a higher risk of atherosclerosis.

The lipid profile of patients with RA has been evaluated in several studies. Some of these studies have reported lower levels of HDL-C and TC, higher serum concentrations of lipoprotein (a) and higher TC/HDL-C and LDL-C/HDL-C ratios in active and/or untreated disease than in the general population [16,20,31,32]. However, other studies have not shown significantly different lipid levels from those observed in the healthy population [33,34] and others refer to an overall reduction in all lipid sub-fractions in cases of active disease [15,35,36]. These contrasting results could be attributed to the size of the samples, the type of study (prospective or cross-sectional), differences in the disease type (established or early), or to differences in the disease activity. Patients in remission or with controlled disease show an increase in HDL-C levels and a reduction in the atherogenic index compared to patients with active disease [15].

Systemic inflammation may also play a role in the development of atherosclerosis [31,33]. In fact, the increase of acute phase reactants in cardiovascular events has already been documented [31]. It has even been suggested that RA and atherosclerosis may share a common predisposition factor [32,33,37]; CRP is the common denominator for both diseases [38,39]. CRP, which increases in active disease, may contribute to atherosclerosis because it stimulates macrophages to produce tissue factor, a procoagulant that is found in atherosclerotic plaques. The presence of CRP in atheromatic lesions also suggests a 'cause and effect' relationship between this acute phase reactant and coronary events [21,39].

An important observation of the present study is that ERA patients exhibit low HDL-C serum levels, which are significantly increased after immuno-intervention in parallel with the reduction of CRP levels and ESR values. Importantly, the increase in HDL-C levels was inversely correlated with the reduction in either CRP levels or ESR values. This suggests that inflammation is an important determinant for the reduced HDL-C levels observed in ERA patients. By contrast, immuno-intervention did not influence the serum levels of LDL-C and triglycerides and it is thus unlikely that inflammation is responsible for the elevation of these lipid parameters observed at baseline in our patients.

In an effort to investigate the biochemical basis for the reduced HDL-C levels exhibited by ERA patients at baseline, we determined the serum levels of CETP activity. CETP exchanges cholesterol esters with triglycerides between HDL and apoB-containing lipoproteins, leading to the reduction in HDL-C levels [21,22]. Increased CETP activity is associated with low HDL-C levels as well as with an overall atherogenic profile [21]. However, several studies have provided contrasting results as to whether CETP itself represents an independent risk predictor for CAD. As HDL-C levels are inversely related to CAD risk, it is hypothesized that inhibition of CETP activity would lead to the reduction in the risk for CAD [40,41]. Based on this assumption, several inhibitors of CETP are currently under investigation in large scale clinical studies [23].

According to our results, ERA patients exhibited significantly higher CETP activity at baseline compared to controls. Thus, we may hypothesize that the reduced HDL-C levels, and consequently the atherogenic lipid profile observed in our patients before treatment, could be attributed at least partially to the higher baseline levels of CETP activity. This hypothesis is further supported by the finding that immuno-intervention significantly reduced CETP activity in parallel with the elevation of the serum HDL-C levels. However, we cannot exclude the possibility that the increased CETP activity and the reduced HDL-C levels observed in our patients at baseline could also be attributed to the fact that several inflammatory mediators inhibit cholesterol efflux from cells by reducing expression of the ATP-binding cassette A1 gene [42]. This possibility is currently under investigation in our laboratory.

The use of DMARDs in ERA for controlling the disease activity may reduce articular damage. Several DMARDs exist for treating RA, but low dose MTX is usually the main choice. Long-term observational studies for periods up to 10 years have shown a sustained clinical response and an acceptable toxicity profile for MTX treatment [43-46]. In addition, patients with severe RA who do not respond to MTX have a poor prognosis, with increased mortality compared to the general population [47], while RA patients who respond to MTX exhibit a substantial survival benefit, mainly by reducing cardiovascular mortality [43,48]. However, there is no clear evidence that the DMARDs used to treat RA decrease the risk of atherosclerosis, or that they are directly protective [49].

Corticosteroids, on the other hand, have a potentially atherogenic effect, given that they cause dyslipidemia and hypertension [15,16]. In spite of this, the effect of their long-term administration in RA is not yet completely understood [15,16]. Several studies have been unable to demonstrate any association between cardiovascular mortality and the use of corticosteroids [50]. A recent study reported a small or no increase in TC levels during long-term administration of corticosteroids [15]. The increase in the prevalence of diabetes mellitus in RA as a late complication of the metabolic syndrome has not been associated with corticosteroid use either [33]. Some authors have extrapolated that the adverse effects of corticosteroids can be counterbalanced, as they exert better control over the inflammatory activity of the disease [51].

It is possible that RA patients may have some classic risk factors for atherosclerosis development. However, it is not correct to attribute the increased prevalence of atherosclerosis observed in RA patients to these factors. In our study, we tried to exclude patients with classic risk factors for atherosclerosis and we found that ERA patients with high disease activity showed an adverse lipid profile before the commencement of therapy. After one year of treatment, a significant clinical and laboratory improvement was noted. This was correlated with changes in the lipid profile, mainly the increase of HDL-C. HDLs are particles with numerous atheroprotective functions, including facilitation of reverse cholesterol transport, improvement of endothelial function, protection of LDL from oxidation, limitation of hemostasis and retardation of inflammatory activity related to the vascular wall [40]. Since our inclusion criteria were very strict, it is obvious that some reservations should be taken for the extrapolation and generalization of our results for all RA patients.

## Conclusion

These findings provide evidence that early immuno-intervention with MTX and corticosteroids controlling the inflammatory process may reduce the risk of atherosclerosis and cardiovascular events in ERA patients. Further, long term longitudinal studies are needed to demonstrate if early treatment in RA patients reduces the risk of cardiovascular events.

## Abbreviations

ACR = American College of Rheumatology; ApoA-I = apolipoprotein A-I; ApoB = apolipoprotein B; CAD = coronary artery disease; CETP = cholesterol ester transfer protein; CRP = C-reactive protein; DAS-28 = disease activity for 28 joint indices score; DMARDs = disease modifying antirheumatic drugs; ERA = early rheumatoid arthritis; ESR = erythrocyte sedimentation rate; HDL-C = high density lipoprotein cholesterol; LDL-C = low-density lipoprotein cholesterol; MTX = methotrexate; RA = rheumatoid arthritis; TC = total cholesterol.

## Competing interests

The authors declare that they have no competing interests.

## Authors' contributions

ANG wrote the paper and participated in the collection of the data. ECP and ESL performed the biochemical and lipoprotein profile. YA carried out the statistics. CK performed the assays for ApoA-I and ApoB. ADT performed the control of the statistics and the lipoprotein profile. AAD conceived the study, participated in its design and coordination and helped to draft the manuscript. All authors read and approved the final manuscript.

## Figures and Tables

**Table 1:** Clinical characteristics and lipid profile of patients with early rheumatoid arthritis and controls

	Controls (*N *= 63)	Patients	
		Baseline (*N *= 58)	Post treatment (*N *= 56)

Sex (male/female)	20/43	14/44	13/43
Age (years)	58.4 ± 17.7	53.6 ± 15.3	54.7 ± 14.8
Body mass index (kg/m^2^)	25.8 ± 17.7	25.5 ± 3.3	25.8 ± 3.1
IgM rheumatoid factor (+/-)	0/0	45/13	44/13
C-reactive protein (mg/dl)	2.1 ± 1.3	28.15 ± 20.75	4.60 ± 4.20^a^
ESR (mm/h)	5.2 ± 3.1	48.0 ± 19.7	14.6 ± 8.7^a^
DAS-28	-	5.8 ± 0.9	2.7 ± 1.0^a^
TC (mg/dl)	190.4 ± 33.9	216.5 ± 50.3^b^	228.1 ± 42.1^c^
LDL-C (mg/dl)	126.5 ± 31.3	141.6 ± 42.3^b^	140.4 ± 32.4
HDL-C (mg/dl)	51.1 ± 7.4	47.5 ± 11.8^2^	60.7 ± 13.4^a^
NonHDL-C (mg/dl)	139.3 ± 30.2	172.0 ± 46.3^b^	167.4 ± 37.4
Triglycerides (mg/dl)	97.1 ± 28.3	133.0 ± 58.2^b^	131.5 ± 56.1
TC/HDL-C	3.7 ± 0.9	4.9 ± 1.3^b^	3.8 ± 0.8^a^
LDL-C/HDL-C	2.5 ± 0.8	3.0 ± 1.0^b^	2.4 ± 0.6^a^
Apolipoprotein B (mg/dl)	93 ± 19	103 ± 28^b^	105 ± 30
Apolipoprotein A-I (mg/dl)	144 ± 23	127 ± 27^d^	152 ± 22^a^

Values represent the mean ± standard deviation. ^a^*p *< 0.001 and ^c^*p *< 0.05 compared to the baseline values; ^b^*p *< 0.001 and ^d^*p *< 0.01 compared to the control group. DAS-28, disease activity for 28 joint indices score; ESR, erythrocyte sedimentation rate; LDL-C, low-density lipoprotein cholesterol; HDL-C, high-density lipoprotein cholesterol; TC, total cholesterol.

## References

[B1] Gravallese EM (2002). Bone destruction in arthritis. Ann Rheum Dis.

[B2] Gabriel SE, Crowson CS, Kremers HM, Doran MF, Turesson C, O'Fallen WM, Matteson EL (2003). Survival in rheumatoid arthritis: a population-based analysis of trends over 40 years. Arthritis Rheum.

[B3] Isomaki HA, Mutru O, Koota K (1975). Death rate and causes of death in patients with rheumatoid arthritis. Scand J Rheumatol.

[B4] Mutru O, Laakso M, Isomaki H, Koota K (1985). Ten year mortality and causes of death in patients with rheumatoid arthritis. Br Med J (Clin Res Ed).

[B5] Watson DJ, Rhodes T, Guess HA (2003). All-cause mortality and vascular events among patients with rheumatoid arthritis, osteoarthritis, or no arthritis in the UK General Practice Research Database. J Rheumatol.

[B6] Pinals RS (1987). Survival in rheumatoid arthritis. Arthritis Rheum.

[B7] Mitchell DM, Spitz PW, Young DY, Bloch DA, McShane DJ, Fries JF (1986). Survival, prognosis, and causes of death in rheumatoid arthritis. Arthritis Rheum.

[B8] Goodson N (2002). Coronary artery disease and rheumatoid arthritis. Curr Opin Rheumatol.

[B9] Van Doornum S, McColl G, Wicks IP (2002). Accelerated atherosclerosis: an extraarticular feature of rheumatoid arthritis. Arthritis Rheum.

[B10] Castelli WP, Garrison RJ, Wilson PW, Abbott RD, Kalousdian S, Kannel WB (1986). Incidence of coronary heart disease and lipoprotein cholesterol levels. The Framingham study. JAMA.

[B11] Kannel WB, Neaton JD, Wentworth D, Thomas HE, Stamler J, Hulley SB, Kjelsberg MO (1986). Overall and coronary heart disease mortality rates in relation to major risk factors in 325,348 men screened for the MRFIT. Multiple Risk Factor Intervention Trial. Am Heart J.

[B12] Manninen V, Elo MO, Frick MH, Haapa K, Heinonen OP, Heinsalmi P, Helo P, Huttunen JK, Kaitaniemi P, Koskinen P (1988). Lipid alterations and decline in the incidence of coronary heart disease in the Helsinki Heart Study. JAMA.

[B13] Cui Y, Blumenthal RS, Flaws JA, Whiteman MK, Langenberg P, Bachorik PS, Bush TL (2001). Non-high-density lipoprotein cholesterol level as a predictor of cardiovascular disease mortality. Arch Intern Med.

[B14] Ross R (1993). The pathogenesis of atherosclerosis: a perspective for the 1990s. Nature.

[B15] Boers M, Nurmohamed MT, Doelman CJ, Lard LR, Verhoeven AC, Voskuyl AE, Huizinga TW, van de Stadt RJ, Dijkmans BA, van der Linden S (2003). Influence of glucocorticoid and disease activity on total and high density lipoprotein cholesterol in patients with rheumatoid arthritis. Ann Rheum Dis.

[B16] Situnayake R, Kitas G (1997). Dyslipidemia and rheumatoid arthritis. Ann Rheum Dis.

[B17] Park YB, Lee SK, Lee WK, Suh CH, Lee CW, Lee CH, Song CH, Lee J (1999). Lipid profiles in untreated patients with rheumatoid arthritis. J Rheumatol.

[B18] Lorber M, Aviram M, Linn S, Scharf Y, Brook JG (1985). Hypocholesterolaemia and abnormal high-density lipoprotein in rheumatoid arthritis. Br J Rheumatol.

[B19] Frati E, Castagna ML, Bacarelli MR, Fioravanti A, Giordano N, Taddeo A, Marcolongo R (1984). Plasma levels of apolipoprotein and HDL-cholesterol in patients with rheumatoid arthritis. Boll Soc Ital Biol Sper.

[B20] Lazarevic MB, Vitic J, Mladenovic V, Myones BL, Skosey JL, Swedler WI (1992). Dyslipoproteinemia in the course of active rheumatoid arthritis. Semin Arthritis Rheum.

[B21] Tall AR (1993). Plasma cholesteryl ester transfer protein. J Lipid Res.

[B22] Barter PJ (2002). Hugh Sinclair Lecture: the regulation and remodelling of HDL by plasma factors. Atheroscler Suppl.

[B23] Forrester SJ, Makkar R, Shah PK (2005). Increasing high-density lipoprotein cholesterol in dyslipidemia by cholesteryl ester transfer protein inhibition. An update for clinicians. Circulation.

[B24] Arnett FC, Edworthy SM, Bloch DA, McShane DJ, Fries JF, Cooper NS, Healey LA, Kaplan SR, Liang MH, Luthra HS (1988). The American Rheumatism Association 1987 revised criteria fort he classification of rheumatoid arthritis. Arthritis Rheum.

[B25] Prevoo ML, van't Hof MA, Kuper HH, van Leeuwen MA, van de Putte LB, van Riel PL (1995). Modified disease activity scores that include twenty-eight-joint counts. Development and validation in a prospective longitudinal study of patients with rheumatoid arthritis. Arthritis Rheum.

[B26] Felson DT, Anderson JJ, Boers M, Bombardier C, Furst D, Goldsmith C, Katz LM, Lightfoot R, Paulus H, Strand V (1995). American College of Rheumatology. Preliminary definition of improvement in rheumatoid arthritis. Arthritis Rheum.

[B27] Tsimihodimos V, Karabina SA, Tambaki AP, Bairaktari E, Miltiadous G, Goudevenos JA, Cariolou MA, Chapman MJ, Tselepis AD, Elisaf M (2002). Altered distribution of PAF-acetylhydrolase activity between LDL and HDL as a function of the severity of hypercholesterolemia. J Lipid Res.

[B28] Friedewald WT, Levy RI, Fredrickson DS (1972). Estimation of the concentration of low-density lipoprotein cholesterol in plasma, without use of the preparative ultracentrifuge. Clin Chem.

[B29] Ordovas JM, Cupples LA, Corella D, Otvos JD, Osgood D, Martinez A, Lahoz C, Coltell O, Wilson PW, Schaefer EJ (2000). Association of cholesteryl ester transfer protein-TaqIB polymorphism with variations in lipoprotein subclasses and coronary heart disease risk. The Framingham study. Arterioscler Thromb Vasc Biol.

[B30] van Gestel AM, Anderson JJ, van Riel PL, Boers M, Haagsma CJ, Rich B, Wells G, Lange ML, Felson DT (1999). ACR and EULAR improvement criteria have comparable validity in rheumatoid arthritis trials. American College of Rheumatology European League of Associations for Rheumatology. J Rheumatol.

[B31] Del Rincon ID, Williams K, Stern MP, Freeman GL, Escalante A (2001). High incidence of cardiovascular events in a rheumatoid arthritis cohort not explained by traditional cardiac risk factors. Arthritis Rheum.

[B32] Dessein PH, Stanwix AE, Moomal Z (2001). Rheumatoid arthritis and cardiovascular disease may share similar risk factors (Letter). Rheumatology.

[B33] Dessein PH, Stanwix AE, Joffe BI (2002). Cardiovascular risk in rheumatoid arthritis versus osteoarthritis: acute phase response related decreased insulin sensitivity and high-density lipoprotein cholesterol as well as clustering of metabolic syndrome features in rheumatoid arthritis. Arthritis Res.

[B34] Hurt-Camejo E, Paredes S, Masana L, Camejo G, Sartipy P, Rosengren B, Pedreno J, Vallve JC, Benito P, Wiklund O (2001). Elevated levels of small, low-density lipoprotein with high affinity for arterial matrix components in patients with rheumatoid arthritis: possible contribution of phospholipase A2 to this atherogenic profile. Arthritis Rheum.

[B35] Munro R, Morrison E, McDonald AG, Hunter JA, Madhok R, Capell HA (1997). Effect of disease modifying agents on the lipid profiles of patients with rheumatoid arthritis. Ann Rheum Dis.

[B36] Svenson KL, Lithell H, Hallgren R, Selinus I, Vessby B (1987). Serum lipoprotein in active rheumatoid arthritis and other chronic inflammatory arthritides. I. Relativity to inflammatory activity. Arch Intern Med.

[B37] Pasceri V, Yeh ET (1999). A tale of two diseases: atherosclerosis and rheumatoid arthritis. Circulation.

[B38] Wallberg-Jonsson S, Cvetkovic JT, Sundqvist KG, Lefvert AK, Rantapaa-Dahlqvist S (2002). Activation of the immune system and inflammatory activity in relation to markers of atherothrombotic disease and atherosclerosis in rheumatoid arthritis. J Rheumatol.

[B39] Jonsson SW, Backman C, Johnson O, Karp K, Lundstrom E, Sundqvist KG (2001). Increased prevalence of atherosclerosis in patients with medium term rheumatoid arthritis. J Rheumatol.

[B40] Gotto AM (2001). Low high-density lipoprotein cholesterol as a risk factor in coronary heart disease: a working group report. Circulation.

[B41] de Grooth GJ, Smilde TJ, van Wissen S, Klerkx AH, Zwinderman AH, Fruchart JC, Kastelein JJ, Stalenhoef AF, Kuivenhoven JA (2004). The relationship between cholesteryl ester transfer protein levels and risk factor profile in patients with familial hypercholesterolemia. Atherosclerosis.

[B42] Baranova I, Vishnyakova T, Bocharov A, Chen Z, Remaley AT, Stonik J, Eggerman TL, Patterson AP (2002). Lipopolysaccharide down regulates both scavenger receptor B1 and ATP binding cassette transporter A1 in RAW cells. Infect Immun.

[B43] Kremer JM, Phelps CT (1992). Long-term prospective study of the use of methotrexate in the treatment of rheumatoid arthritis. Update after a mean of 90 months. Arthritis Rheum.

[B44] Papadopoulos NG, Alamanos Y, Papadopoulos IA, Tsifetaki N, Voulgari PV, Drosos AA (2002). Disease modifying antirheumatic drugs in early rheumatoid arthritis: a longterm observational study. J Rheumatol.

[B45] Sany J, Anaya JM, Lussiez V, Couret M, Combe B, Daures JP (1991). Treatment of rheumatoid arthritis with methotrexate: a prospective open longterm study of 191 cases. J Rheumatol.

[B46] Weinblatt ME, Weissman BN, Holdsworth DE, Fraser PA, Maier AL, Falchuk KR, Coblyn JS (1992). Long-term prospective study of methotrexate in the treatment of rheumatoid arthritis. 84-month update. Arthritis Rheum.

[B47] Krause D, Schleusser B, Herborn G, Rau R (2000). Response to methotrexate treatment is associated with reduced mortality in patients with severe rheumatoid arthritis. Arthritis Rheum.

[B48] Choi HK, Hernan MA, Seeger JD, Robins JM, Wolfe F (2002). Methotrexate and mortality in patients with rheumatoid arthritis: a prospective study. Lancet.

[B49] Rewald E, de las Mercedes Francischetti M (2002). Methotrexate treatment and mortality in rheumatoid arthritis. Lancet.

[B50] Wallberg-Jonsson S, Ohman ML, Dahlqvist SR (1997). Cardiovascular morbidity and mortality in patients with seropositive rheumatoid arthritis in Northern Sweden. J Rheumatol.

[B51] Park YB, Choi HK, Kim MY, Lee WK, Song J, Kim DK, Lee SK (2002). Effects of antirheumatic therapy on serum lipid levels in patients with rheumatoid arthritis: a prospective study. Am J Med.

